# The Role of the Cognitive Control System in Recovery from Bilingual Aphasia: A Multiple Single-Case fMRI Study

**DOI:** 10.1155/2016/8797086

**Published:** 2016-11-14

**Authors:** Narges Radman, Michael Mouthon, Marie Di Pietro, Chrisovalandou Gaytanidis, Beatrice Leemann, Jubin Abutalebi, Jean-Marie Annoni

**Affiliations:** ^1^Neurology Unit, Department of Medicine, Faculty of Sciences, University of Fribourg, Fribourg, Switzerland; ^2^Neurorehabilitation Department, University Hospital, University of Geneva, Geneva, Switzerland; ^3^Neuropsychology Unit, Fribourg Cantonal Hospital, Fribourg, Switzerland; ^4^Center for Neurolinguistics and Psycholinguistics, San Raffaele University and Scientific Institute San Raffaele, Milan, Italy

## Abstract

Aphasia in bilingual patients is a therapeutic challenge since both languages can be impacted by the same lesion. Language control has been suggested to play an important role in the recovery of first (L1) and second (L2) language in bilingual aphasia following stroke. To test this hypothesis, we collected behavioral measures of language production (general aphasia evaluation and picture naming) in each language and language control (linguistic and nonlinguistic switching tasks), as well as fMRI during a naming task at one and four months following stroke in five bilingual patients suffering from poststroke aphasia. We further applied dynamic causal modelling (DCM) analyses to the connections between language and control brain areas. Three patients showed parallel recovery in language production, one patient improved in L1, and one improved in L2 only. Language-control functions improved in two patients. Consistent with the dynamic view of language recovery, DCM analyses showed a higher connectedness between language and control areas in the language with the better recovery. Moreover, similar degrees of connectedness between language and control areas were found in the patients who recovered in both languages. Our data suggest that engagement of the interconnected language-control network is crucial in the recovery of languages.

## 1. Introduction

Due to the increasing number of multilinguals in modern society, the incidence of language impairments induced by brain lesions (aphasia) in this population is growing rapidly [1, 2]. The rehabilitation of multilingual aphasic patients represents an important challenge for clinicians because (i) since the representation of first (L1) and second (L2) languages partly overlaps in bilinguals' brains, brain lesions do not necessarily affect L1 and L2 equally [3]; and (ii) recovery patterns for each language in multilingual aphasic patients vary considerably and so far are unpredictable [4].

Most of the current literature indicates that language recovery in bilingual aphasic patients depends on the degree of language mastery or language-specific factors [5–7]. For example, similarities in typology, phonological, morphological, lexical, and syntactic aspects between languages are shown to affect the pattern of recovery of languages in bilingual aphasic patients [1, 6]. Such an approach is also supported by evidence that changes in second language expertise and use are associated with an increase of connectivity within the language network of healthy subjects. However, growing evidence suggests that the control system may also play a key role in this process [5, 8, 9]. In healthy bilingual speakers, cognitive control system is strongly involved in language production [10] because language representations must be manipulated and monitored both within the language being spoken and across languages to select the appropriate vocabulary and syntax and to inhibit the nontarget language [11].

Abutalebi and Green [10], for instance, propose a “dynamic view” in which the pattern of language recovery in bilingual aphasia depends on the patient's ability to select and control language activation [10, 12]: (i) a parallel recovery, in which both impaired languages improve to a similar extent, and, concurrently, occurs when both languages are inhibited to the same degree; (ii) an antagonistic recovery, in which the patient is able to speak in one language on one day while on the next day only in the other, occurs when inhibition affects only one language for a period of time and then shifts to the other language (with disinhibition of the previously inhibited language); (iii) a selective recovery, in which one language remains impaired while the other recovers, occurs if the lesion has permanently raised the activation threshold for one language; and (iv) a pathological mixing, in which the elements of the two languages are involuntarily mixed during language production, occurs when languages can no longer be selectively inhibited [9, 10, 13].

While this theory accounts for the large variability in recovery patterns of multilingual aphasia, there is only sparse evidence for any association between control function and language recovery since control functions are rarely specifically assessed in aphasic patients. Aglioti et al. [5] reported the case of a bilingual aphasic patient who showed a greater deficit in her more used L1 than in her less practiced L2, following lesions mainly involving the left basal ganglia. The authors suggest that the patient's deficit in L1 may be considered as a pathological fixation on a foreign language resulting from a deficit in switching between languages. However, the patient had a normal performance in the Wisconsin card-sorting test (WCS), a nonverbal task testing the ability to change from one criterion of choice to another. This result suggested that, in the absence of a remarkable impairment in control functions (shown in WCS which evaluated “shifting,” a part of control functions), the patient's fixation behavior was mostly linguistic. Moreover, since the assessment of executive functions was conducted one year after the stroke, anatomofunctional plastic reorganization of the language and control networks could already have taken place and likely confounded the results. An earlier evaluation (e.g., at acute or subacute phase) following the stroke could have better shown whether this so-called pathological fixation on L2 and the L1 impairment has resulted from impairment in cognitive control function. Verreyt et al. [14] reported the case of an early French-Dutch bilingual aphasic who, following a lesion to the left thalamus, presented larger impairment in Dutch. By showing cognate facilitation and cognate interference effects in different lexical decision tasks and an impaired performance in the flanker task, the authors suggested that the differential pattern of impairment in language could be explained by a language-control deficit. In addition, Abutalebi et al. [9], in a longitudinal, single-case study of a chronic bilingual aphasic patient combining fMRI and dynamic causal modelling (DCM), showed an increased connectivity within the control and language networks for the treated and recovered language. In line with the Paradis's activation threshold theory, which holds that lesions that do not completely damage language areas but cause an imbalance in activating and inhibiting languages are responsible for aphasia in bilinguals [12], they found that the engagement of the areas mediating language control played a crucial role in language recovery in bilingual aphasic patients. They showed that connections between language and control areas were stronger in the language that recovered better, probably because it received more resources for its functioning.

The network underlying language control described by Abutalebi and Green [10] and Abutalebi et al. [9] includes the prefrontal cortex (mainly inferior prefrontal cortex including LIFGOrb (left inferior frontal gyrus pars orbitalis, BA47)), the anterior cingulate cortex (ACC) (BAs24, 32, 33), and the basal ganglia. This network is interconnected with language areas involved in word production (LIFGTri: left inferior frontal gyrus pars triangularis, BA45) and “basal temporal language area, BTLA” involved in semantic decoding during picture naming (posterior part of the left inferior temporal gyrus BAs19 and 37). In the bilingual brain, the prefrontal cortex is involved in word production in the less proficient language and in inhibiting responses from the more proficient language. Together with the anterior cingulate that detects response conflicts, it constitutes a control loop in which the identification of conflict triggers a top-down signal from the prefrontal cortex to modulate the nontarget representation (see [10, 15, 16]). The left caudate and the ACC are strongly connected to the prefrontal cortex [17] and work together with this structure to inhibit interferences from the nontarget language. The ACC signals potential response conflicts or errors to the prefrontal cortex (i.e., in the case that an erroneous language has been chosen) and the prefrontal cortex then seeks to avoid incorrect selection. Finally, the basal ganglia may subserve language planning, that is, the activation of a given language as a main function of the left caudate and the control of articulatory processes in the left putamen (see [18, 19]). Using linguistic and nonlinguistic switching tasks, it has been shown that the neuroanatomical bases of language control and domain-general cognitive control share the partially overlapping structures, although their involvement may vary [20, 21]. It is worth noting that understanding neural mechanisms underlying patterns of recovery has many implications for the therapeutic approach.

Based on the hypothesis of a key role for cognitive control in bilingual language production and in the recovery of bilingual aphasia, our study aims to test whether among the different control areas proposed by Abutalebi et al. [9], changes in certain connections between control and language areas influence the recovery of language (namely, between LIFGTri and LIFGOrb and LC and ACC). To this aim, we tested five late bilingual patients who suffered from aphasia following a focal left-hemispheric brain lesion. The patients were evaluated at two time points (subacute and chronic phases, three months apart). Three main analyses were conducted to examine the pattern of changes in patients' language and control functions, connectivity within language-control network, and possible correlation between behavioral performances and connectivity with language-control network.

- As a descriptive marker of behavioral improvement/changes in language and control functions, the patients were behaviorally evaluated for their pattern of recovery of language and executive functions using general aphasia evaluation (GAE), picture naming and executive tasks (linguistic and nonlinguistic switching).
- In order to investigate the connections within the language-control network, we used fMRI analyses and applied dynamic causal modelling in the fMRI picture naming task in L1 and L2 to examine whole brain activation patterns and the effective connectivity between the control areas (ACC, left caudate nucleus, and LIFGOrb) and the regions involved in language production (especially LIFGTri). We further examined whether global changes in connectedness within language-control network are associated with the recovery of languages.
- To directly assess the hypothesis advanced in the language-control model [9], we examined the correlations between the recovery of language functions and the changes in the strength of connections between the above-mentioned areas using group analyses. In fact, as Meier et al. [22] in a DCM study on chronic aphasic patients and a group of controls have found that language network parameters are specifically associated with naming abilities in picture naming task, we consider that there should be a difference in connection strength in L1 and L2 and also according to naming improvement across time.

We chose to evaluate bilingual aphasic patients during the subacute phase since this population has rarely been studied in the acute and subacute phases. This will allow us to better understand the contribution of the control system in the recovery of language in bilingual aphasia, especially during the period when spontaneous recovery process mainly takes place [6, 23]. In addition, in this phase, the spontaneous recovery and neural plasticity processes are ongoing and given that bilingual population is strongly relied on cognitive control system, we assume that the changes in cognitive control system and its interconnection with the language system probably play a role in the recovery of aphasia.

## 2. Methods

### 2.1. Participants

#### 2.1.1. Aphasic Patients

We recruited right-handed late (age of acquisition (AoA) of L2 after 6 y/o) bilingual patients aged between 18 and 85 years old, who suffered from aphasia following a focal left-sided ischemic or hemorrhagic stroke. The following languages were included in the study: French (in each case as subjects' L1 or L2) and English, German, Spanish, or Italian. During the recruitment procedure, we excluded patients with a history of premorbid language impairment, several brain lesions, or severe aphasia.

A total of eleven patients were recruited for this project. However, only six patients completed all the steps of the study and, among them, five subjects fulfilled our criteria of the selection of regions of interest (ROIs) for the DCM analyses and therefore are reported in this paper. Five more patients performed the first session of the study and then declined to participate in the second session (see Section 2.3 for details of the steps of the study) and were therefore not included in the analyses. Among the five patients included in this paper (aged 61.6 (±6.9) years old and including two females), three patients were French (L1) and English (L2) and two patients were Italian (L1) and French (L2). All the patients were late bilinguals (AoA: 16.5 ± 5.1). The lesion of each patient is shown in a figure specifically designed for each of them (Figures 3(a) [Fig fig4] [Fig fig5] [Fig fig6]–7(a)). The study procedure was approved by the local Ethics Committees of Geneva University Hospital (CE 12-274) and Fribourg Cantonal Hospital (018/12-CER-FR). 

*Case Description*

*Patient 1.* YL is a 61-year-old man who is a French (L1)-English (L2) bilingual. Mr. YL was born in French-speaking part of Switzerland. The language of teaching at school was French. Mr. YL estimates an advanced level of English for reading, speaking, and comprehension (all between 95 and 100% according to the self-evaluation scale of L2 level). Before the stroke, his language usage was mainly in French; he spoke 100% French with his family and 80% with his friends. He followed TV and radio programs only in French. However, his reading was 50% in French and 50% in English (readings in English are mainly work-related books and documents), and he used mainly English at his workplace (80%).

Mr. YL was admitted to Geneva University Hospital (HUG) with right sensorimotor hemiparesis, right facial palsy, and impaired comprehension and language production mainly manifested in L2 following a left frontotemporal ischemic stroke. A secondary hemorrhagic event in the ischemic area was seen three days after the ischemic event (Figure 3(a)). A first language evaluation showed a transcortical sensory aphasia; he presented mainly auditory comprehension problems and produced repeated semantic errors. However, spontaneous speaking was relatively fluent.

*Patient 2.* MR is a 65-year-old Italian (L1)-French (L2) bilingual woman. She was born in Italy to Italian parents and followed primary school in Italy. She moved to the French-speaking part of Switzerland at the age of 24, and then she has taken some courses to learn French. Before the stroke, she used Italian and French quite equally; she used French at work (100%), and Italian for TV or radio programs (100%). She used 50% in Italian and 50% in French to speak with her family and friends and to read books and journals.

MR was admitted to HUG for resection of a meningioma on the left greater wing of the sphenoid bone. Two days after the resection of the meningioma, she presented a right sensorimotor hemiparesis and a severe language production problem plus a lesser degree of comprehension problems in both languages, caused by an epidural hematoma with pressure over the operation site and ischemic changes in the left frontobasal area (Figure 4(a)). The initial language evaluation showed anomia in both L1 and L2.

*Patient 3.* CA is a 63-year-old woman who is an Italian (L1)-French (L2) bilingual. She was born in Italy to Italian parents and followed primary school in Italy. She moved to the French-speaking part of Switzerland at the age of 10; thereafter she started to learn French. Mrs. CA followed secondary school in Switzerland where the teaching language was French. She has also basic knowledge in English and Spanish, which she has learned at school. Before the stroke, the main language of conversation was French with her husband and children (90%) and at work (75%) and she spoke Italian with her parents (100%).

She was admitted to HUG because of right hemiparesis and severe global aphasia due to a left basal ganglia hemorrhagic stroke with no evidence of midline shift (Figure 5(a)). Within a few days, global aphasia developed into severe anomia with hypophonia mainly affecting L2.

*Patient 4*. RG is a 49-year-old bilingual French (L1)-English (L2) male patient. He finished primary and secondary schools in the French-speaking part of Switzerland. He started to learn English at school at the age of 14. He used English quite frequently in his daily life; he used French and English equally at work (50% French and 50% English for teaching and customer care). He followed TV and radio programs and also read books and journals 50% in French and 50% in English. However, he spoke only in French with his friends and family. According to the self-evaluation questionnaire filled in by his wife, his language abilities were estimated as follows: speaking 50%, comprehension 70%, reading 85%, and writing 30%.

He was admitted to Fribourg Cantonal Hospital with a sudden right hemiparesis and anomia and no other language symptoms due to a left sylvian ischemic stroke (Figure 6(a)).

*Patient 5.* GH is a 79-year-old bilingual French (L1)-English (L2) male patient. He has learned English around the age of 18 when he first travelled to the US and England. He has then moved to Sweden and started to learn Swedish too. He has been working in Sweden for about 18 years teaching guitar in both English and Swedish. He then moved back to Switzerland at the age of 66. He then continued to teach playing guitar to children. He used both French and English in his teachings (50% French and 50% English). With his family he spoke only in French; however with his friends he spoke 50% in French and 50% in English. He followed TV and radio program mostly in French (75% in French and 25% in English) and he read books and journals only in French.

He was admitted to HUG with a paresthesia in his left arm and global aphasia. GH was a known case of auricular fibrillation before this acute event. The cerebral CT scan after the acute event confirmed an ischemic lesion in the left frontal, insula, and sylvian areas (Figure 7(a)). Within a few days, global aphasia developed into severe anomia and increased switching behavior.

More details of patients can be found in Tables 1 and 2 and Supplementary Data 1 available online at http://dx.doi.org/10.1155/2016/8797086.

#### 2.1.2. Control Subjects

The data and results on the control subjects are presented in Supplementary Data 2 and 3.

### 2.2. Assessment of Premorbid Language Proficiency

Subjects were assessed using a questionnaire on their immersion in both L1 and L2, AoA, how long they had lived in a region where predominantly the second language was spoken, which language they spoke with their family members, in school, and in present activities (watching TV/listening to radio, reading books, and mental arithmetic), and if the language was acquired in school or out of school only. In the self-evaluation part, subjects (or their family members) had to indicate in percentages how well they would estimate their reading, speaking, comprehension, and writing skills.

### 2.3. Study Design

Patients were assessed at subacute (three to five weeks after stroke onset, T1) and chronic (three months after T1 evaluation, T2) phases. In both sessions we used the same procedures, listed as follows: (1) behavioral assessment of the severity of aphasia as well as a combination of language-control function evaluations; (2) in an fMRI recording session, the patients performed a language production task (picture naming) in each language (see Section 2.6.1 for picture naming task).

### 2.4. Behavioral Tasks

General aphasia evaluation (GAE): global severity of the aphasia and language capacities was assessed using a separate evaluation of language capacities in each language (i.e., L1 and L2 were evaluated separately, one day apart). This evaluation consisted of a brief test of object naming (ten objects to name), automatic speech (series: days of the week, counting from 1 to 25), word and phrase repetition, yes/no questions, object recognition, following oral and written instructions (simple, semicomplex, and complex commands), description, and verbal fluency. All these tests were extracted from the Bilingual Aphasia Test (BAT) [24] except for yes/no questions which were extracted from the Mississippi Aphasia Screening Test (MAST) [25]. This evaluation material has been already used in our previously published works, for example, [26]. As a result, a production index of maximum 52 scores (i.e., the sum of the scores obtained from production tasks including object naming, series, verbal fluency, word and phrase repetition, and description) and a total score (maximum 96 scores) was obtained.

**Figure 1:**
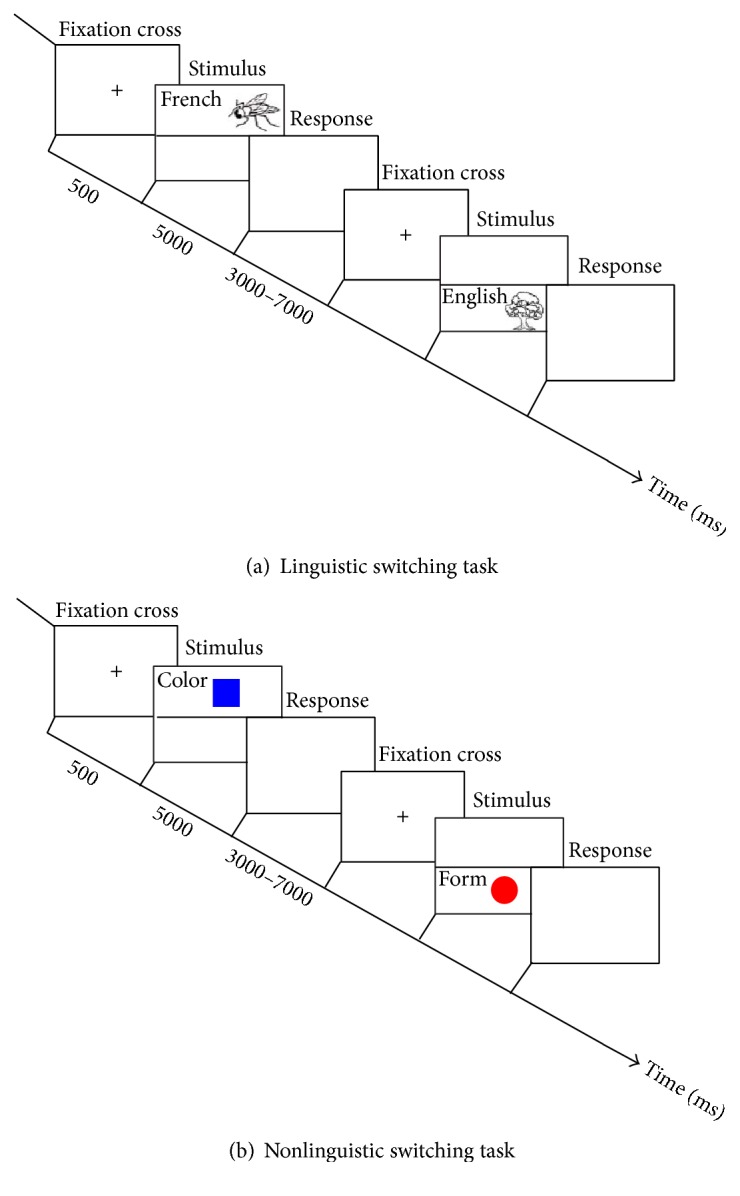
(a) Linguistic switching task. This task includes 40 trials. Only the six first trials were cued; the language in which the image should be named (L1 or L2) is written in the left of the image. (b) Nonlinguistic switching task. This task includes 40 trials. Only the six first trials were cued; the category in which the image should be named (color or form) is written in the left of the image.

Language-control functions were evaluated using the following:

- A linguistic switching task (adapted from Abutalebi et al. 2008 for aphasic patients [27]): forty images (black and white line drawing picture) of Snodgrass and Vanderwart [28] (all noncognate words) were used for each list. Eight pairs of lists were prepared (a combination of French as first or second language and the other four languages). The words of each pair were matched for word frequency. The subjects were asked to name, as quickly as possible, the images in L1 when the image appeared on the upper part and name the image in L2 when the image appeared on the lower part of the screen. After a fixation cross of 500 ms, the images were presented on the screen for 5,000 ms and were followed by a blank screen of variable duration of 3,000–7,000 ms (to provide a random duration of the interstimulus interval). Therefore, the subjects had at most between 8,000 and 12,000 ms to respond. However, only first-attempt correct responses within five seconds of the presentation of the image were scored as correct. Each trial was started manually by the experimenter when the word “ready?” was presented on the screen. The first six trials of the task were cued with the language in which the image should be named (L1 or L2) written on the left of the image (Figure 1(a)). This task lasted between 10 and 12 minutes depending on patients' response time.
- A nonlinguistic switching task: four images (a red or blue circle or square) were presented on the upper or the lower part of the screen. Subjects were instructed to name, as quickly as possible, the color of the image when the image was presented on the upper part of the screen and to say the shape of the image when it was presented on the lower part of the screen. After a fixation cross of 500 ms, the images were presented on the screen for 5,000 ms and were followed by a blank screen of variable duration of 3,000–7,000 ms (to provide a random duration of the interstimulus interval). Therefore, the subjects had at most between 8,000 and 12,000 ms to respond. However, only first-attempt correct responses within five seconds of the presentation of the image were scored as correct. Each trial was started manually by the experimenter when the word “ready?” was presented on the screen. The first six trials of the task were cued with the category in which the image should be named (color or shape) written on the left of the image (Figure 1(b)). The task lasted around 10–12 minutes depending on patient's response time.

For all the tasks, instructions were given both written on the screen and orally, and the subjects performed a short training session just before starting the task. The evaluation of the language-control function was performed in the more proficient language (usually L1). Moreover, because of slowness of patients and fatigability, for all the tasks we did not record reaction times, and the analyses were focused on response accuracy.

### 2.5. Behavioral Data Analyses

Because of the limited number of patients, differences in lesion size and site, and variability of symptoms, we used primarily a multiple single-case approach for our analyses between T1 and T2. For comparison of the patients' scores in the two sessions, a McNemar Chi-squared test is used for each case. GAE, picture naming, and “combined production score” (i.e., the average response accuracy percentage in picture naming and production score of the GAE) are assessed as language performances. Specifically, we focused on the “combined production score” which could better represent language production performance.

### 2.6. Functional Magnetic Resonance Imaging Task

#### 2.6.1. Picture Naming in L1 and L2

*Stimuli.* Five lists (one list per language) of 40 noncognate words (black and white line drawing pictures) were selected from Snodgrass and Vanderwart [28]. The words were matched for word frequency across all the lists.

*Procedure.* Each fMRI session started with a picture naming in L1; in this part, the subjects were instructed to name the pictures that appeared on the screen in their L1. After a fixation cross of 500 ms, the images were presented on the screen for 5,000 ms and were followed by a blank screen of variable duration of 4,100–6,100 ms (to provide a random duration of the interstimulus interval). Therefore, the subjects had at most between 9,100 and 11,100 ms to respond. However, only first-attempt correct responses within five seconds of the presentation of the image were scored as correct. Each task lasted around 7-8 minutes (a total of around 15 minutes for picture naming in both L1 and L2). After about 30 seconds of rest, the subjects started their second task in which they had to name the pictures in their second language. The first six trials of the task were cued with the language in which the image should be named (L1 or L2) written on the left of the image. For the fMRI tasks, a short training was performed before the subjects entered the scanner. In this training, which contained 10 trials, the subjects were presented with black and white line drawing pictures selected from Snodgrass list and were asked to name the pictures in their L1 or L2 to become familiar with the task.

### 2.7. FMRI Acquisition

Data of the aphasic patients were acquired using three different 3T scanners on two different sites; Site 1: Fribourg Cantonal Hospital (HFr) and Site 2: University Hospital of Geneva (HUG). The scanners which were used were (1) Discovery MR750; GE Healthcare, Waukesha, Wisconsin, with a 32-channel receive head coil (Site 1), (2) Magneton Trio, Siemens Medical Solutions, Erlangen, Germany, with a 12-channel receive head coil (Site 2), and (3) Magneton Prisma, Siemens Medical Solutions, Erlangen, Germany, with a 20-channel receive head (Site 2). Subjects were in a supine position with their heads stabilized by foam to reduce head movements. They wore headphones (MKII system from MR confon, Magdeburg, Germany) coupled with an MRI-compatible microphone (FOMRI-III system from Optoacoustics, Israel) to record oral response during the experiment. In the first scanner, visual stimuli were presented on an LCD screen (NordicNeuroLab, Bergen, Norway). In the other two scanners, the stimuli were displayed on a screen by a video projector (Hitachi CP-X1200 with long focal distance Hitachi LL-504, Hitachi Ltd., Tokyo, Japan) through a mirror system. In all three cases, the stimuli resolution was 1024 × 768 with a refresh rate of 60 Hz. The E-Prime 2 software (Psychology Software Tools, Pittsburgh, USA) was used to show stimuli and record behavioral data.

### 2.8. MRI Acquisition

MRI acquisition parameters were optimized for each site. From the first site in Fribourg (Scanner 1), T1-weighted images were acquired with a FSPGR BRAVO sequence, voxel size: 0.86 × 0.86 × 1 mm, field of view (FOV) = 220 mm, number of coronal slices: 276, TR/TE = 7300/2.8 ms, flip angle = 9, phase acceleration factor (PAF) = 1.5, and intensity correction (SCIC). Functional T2^*∗*^weighted echo planar images (EPI) with blood oxygenation level-dependent (BOLD) contrast were acquired with voxel size: 2.3 × 2.3 × 3 mm, FOV = 220 mm, 37 ascending axial slices, interslice spacing = 0.2 mm, TR/TE = 2000/30 ms, flip angle = 85, and PIAF: 2. In addition, a B0 field inhomogeneity mapping sequence was acquired to correct for geometrical distortion that occurred along the phase-encoding direction (using a Gradient Echo protocol) with the same scan coverage as the functional scan: number of slices = 37, FOV = 220 mm, TR/TE_1_/TE_2_ = 50/4.9/7.3 ms [29]. From the second site (scanners 2 and 3), T1 weighted images were acquired with an MP Rage sequence, voxel size: 0.86 × 0.86 × 1.1 mm, FOV = 220 mm, number of coronal slices: 208, TR/TE = 2500/2.94 ms for scanner 2 and 2500/2.97 for scanner 3, flip angle = 9, and PAF: 2. Functional T2^*∗*^weighted EPI with BOLD contrast were acquired with voxel size: 2 × 2 × 3.5 mm, FOV = 240 mm, 29 ascending axial slices, interslice spacing = 0.35 mm, TR/TE = 2000/30 ms, flip angle = 85, and PIAF: 2. A B0 field inhomogeneity mapping sequence was also acquired with the same scan coverage as the functional MRI sequences: number of slices = 29, FOV = 240 mm, and TR/TE_1_/TE_2_ = 400/5.19/7.65 ms. On average, a total of 248 volumes were acquired during the picture naming in L1 and picture naming in L2. Each fMRI acquisition session started with six seconds of dummy scans to ensure a steady-state magnetization of the tissues.

### 2.9. Functional MRI Preprocessing

We used the SPM8 software (Welcome Trust Centre for Neuroimaging, Institute of Neurology, University College London), running on MATLAB 2012b (MathWorks, Inc., MA, USA), to analyze functional MRI data (fMRI). FMRI images were preprocessed following the standard procedure proposed by Friston [30]. Preprocessing steps included a spatial realignment, unwrapping (using the FieldMap 2.1 toolbox [31]), slice timing (with middle temporal slice as reference), coregistration on T1 image, normalization on the Montreal Neurological Institute (MNI) space with 3 × 3 × 3 mm^3^ voxel size, and smoothing with a Gaussian kernel of 8 mm full width at half maximum (FWHM). In order to exclude the brain lesion from the analyses, a mask file of the brain lesion of each subject was manually drawn on axial slices of the standard Montreal Neurological Institute's (MNI) brain template using the MRIcron software (https://www.nitrc.org/projects/mricron) and used during the preprocessing of data on SPM. The preprocessed volumes were submitted to fixed effects analyses at the subject level by applying the general linear model to each voxel [32]. Each stimulus onset was modelled as an event encoded in condition-specific “stick-functions” and convolved with a canonical hemodynamic response function. A separate model was built for picture naming in L1 and picture naming in L2. In addition, movement parameters were included as regressors of no interest. Time series from all voxels were submitted to a high-pass filter with a 1/250 Hz threshold, and an autoregressive function (AR (1)) was applied.

### 2.10. Dynamic Causal Modelling (DCM)

DCM is a widely used method for investigating context-dependent causal interactions between brain regions and it describes the architecture of the network (i.e., the ROIs and the connections). In DCM, the brain is treated as a dynamic input-state-output system. A given experiment is considered as a designed perturbation of neuronal dynamics that is propagated throughout a network of interconnected nodes. Three sets of parameters are estimated in DCM: the direct influence of stimuli on regional activity (driving input), the intrinsic connections between regions, and the changes in the intrinsic connectivity between regions induced by adding or removing a modulatory influence (modulatory effect).

**Figure 2:**
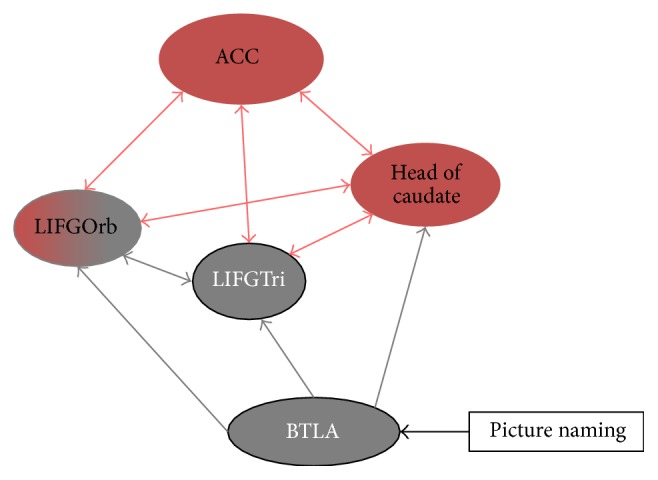
The structure of language-control network. This network was proposed by Abutalebi et al. (2009). Connections between brain areas involved in picture naming (in black) and control (in red). The modulatory effect of the experimental task (picture naming in L1 and L2) was added to the model on BTLA. ACC: anterior cingulate cortex, LIFGTri: left inferior frontal gyrus-pars triangularis, LIFGOrb: left inferior frontal gyrus-pars orbitalis, BTLA: basal temporal language area.

We based our analyses on this model, which has been defined by Abutalebi et al. [9]. Because of the variability of lesion site in our patients, in order to be able to compare changes in connectivity with the same model across all subjects/conditions, we have defined this model for all subjects and conditions. We have not selected a fully connected model (i.e., with all possible connections within the network) to avoid having a very complex model and overfitting of the data. In addition the structure of this model was designed based on* a priori *hypotheses already tested by previous works. Therefore, the selection of the ROIs and the intrinsic connections was based exactly on the work by this group. Accordingly, the following five ROIs were selected for the network: BTLA, LIFGTri (areas related to language processing), head of left caudate, ACC (areas involved in cognitive control function), and LIFGOrb as a part of both language and cognitive control systems. As per Abutalebi et al. [9], we also included only left-hemispheric regions as our main focus was the effect of control areas on the intrahemispheric reorganization of language areas (see Figure 2 for the model structure). The same model was used for all subjects (patients and controls) and for both testing sessions. Using TD-ICBM-MNI template atlas, we prepared the mask for the ROIs. Individual subject time series data from each subject's individual activation map threshold at *p* < 0.05 uncorrected were extracted from each 7 mm spherical ROI centered at the subject's local maximum inside the ROIs. For the patients, we have verified visually whether the ROIs were affected by the lesion. When the patients or control subjects did not fulfill our criteria (showing activation with threshold < 0.05 uncorrected in all 5 ROIs and/or absence of lesion in the ROIs) they were removed from the analyses. This way, we have removed one patient (as one of the ROIs was inside the lesion) and one control participant (as he did not show activation in one of the ROIs in the desired threshold) [33].

However, in order to take into account the modulatory effect of the language task on the network (which was not included previously in the model by Abutalebi et al.), we inserted the modulatory effect of the task over BTLA (as the sensory input of the network) [33] and LIFGTri as two different models. We compared the three models (two models with modulatory effect of picture naming on LIFGTri or BTLA and a model with no modulatory effect) using a Bayesian model selection with a fixed effect strategy which assumes that the optimal structure is assumed to be identical across subjects, and the model with modulatory effect over the BTLA best explained fMRI activation through the different patients and controls (separately) during the naming in L1 and L2 according to this comparison. We therefore employed this model in all our subjects. The DCM model was deterministic, bilinear with one state per region. The analyses of DCM were performed using SPM12 and using the data preprocessed in SPM8.

### 2.11. FMRI Data Analyses

**Figure 3:**
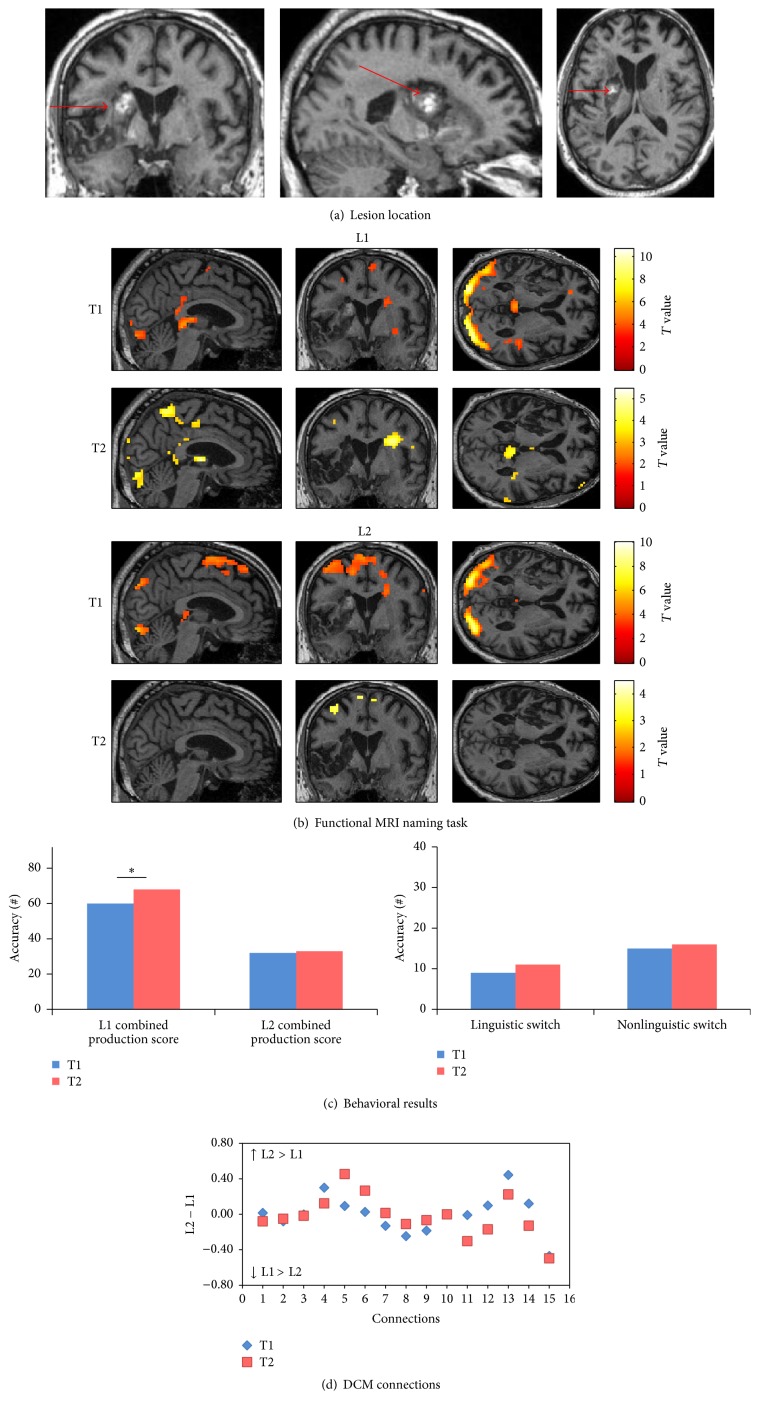
Patient 1. (a) Ischemic stroke in left frontotemporal area in the T1-weighted MRI image at T1. (b) Pattern of brain activation in different conditions while picture naming, with an uncorrected *p* < 0.001 for the main effects. (c) Behavioral results of the combined production scores in both languages, linguistic and nonlinguistic switching scores across sessions. *∗* represents *p* value < 0.05. (d) Differences between L1 strength values and L2 strength values for each single connection across sessions. (1) ACC to LIFGTri. (2) ACC to LIFGOrb. (3) ACC to LC. (4) LC to ACC. (5) LC to LIFGTri. (6) LC to LIFGOrb. (7) LIFGTri to LC. (8) LIFGTri to ACC. (9) LIFGOrb to LC. (10) LIFGOrb to ACC. (11) LIFGTri to LIFGOrb. (12) LIFGOrb to LIFGTri. (13) BTLA to LIFGTri. (14) BTLA to LIFGOrb. (15) BTLA to LC.

**Figure 4 fig4:**
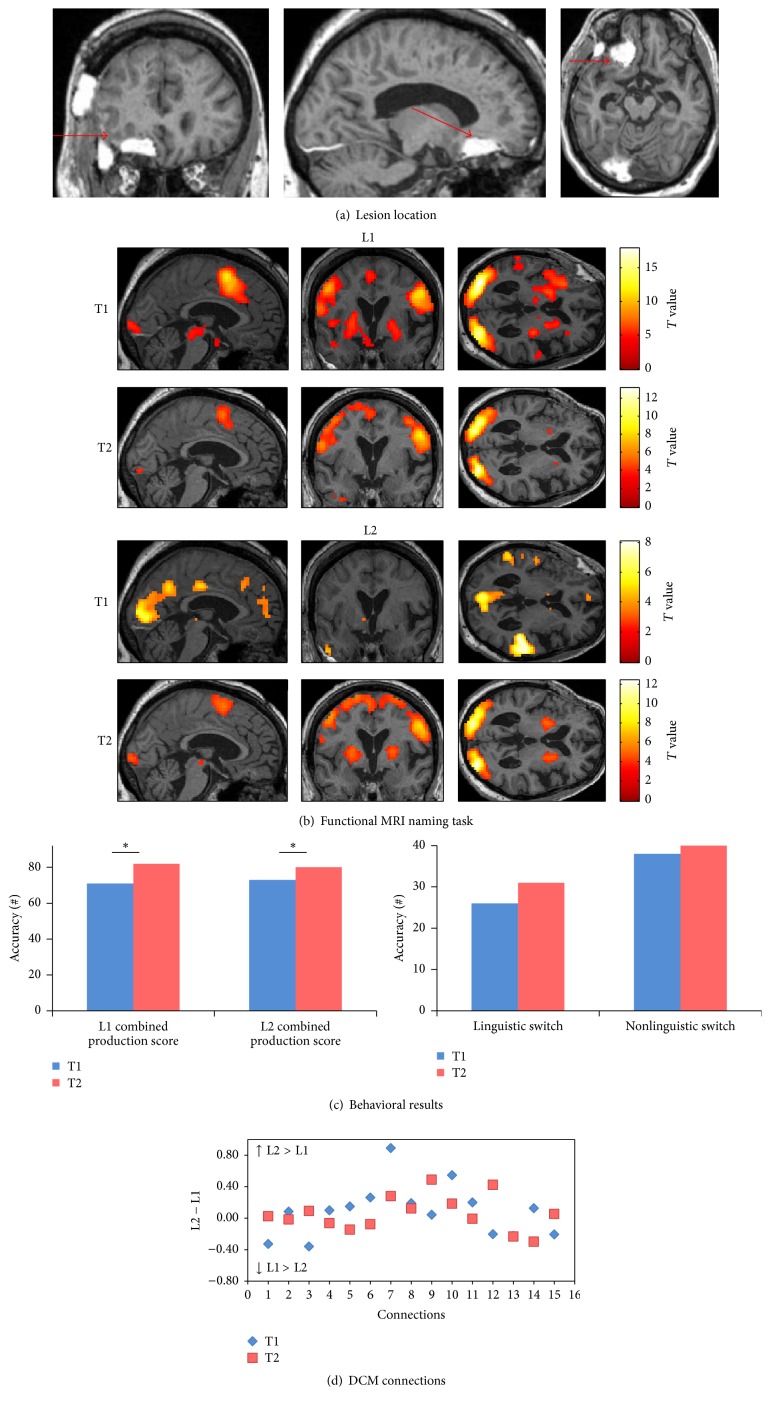
Patient 2. (a) The T1-weighted MRI image at T1 shows an epidural hematoma with pressure over the operation site on the left frontobasal area. (b) Pattern of brain activation in different conditions while picture naming, with an uncorrected *p* < 0.001 for the main effects. (c) Behavioral results of the combined production scores in both languages, linguistic and nonlinguistic switching scores across sessions. *∗* represents *p* value < 0.05. (d) Differences between L1 strength values and L2 strength values for each single connection across sessions. (1) ACC to LIFGTri. (2) ACC to LIFGOrb. (3) ACC to LC. (4) LC to ACC. (5) LC to LIFGTri. (6) LC to LIFGOrb. (7) LIFGTri to LC. (8) LIFGTri to ACC. (9) LIFGOrb to LC. (10) LIFGOrb to ACC. (11) LIFGTri to LIFGOrb. (12) LIFGOrb to LIFGTri. (13) BTLA to LIFGTri. (14) BTLA to LIFGOrb. (15) BTLA to LC.

**Figure 5 fig5:**
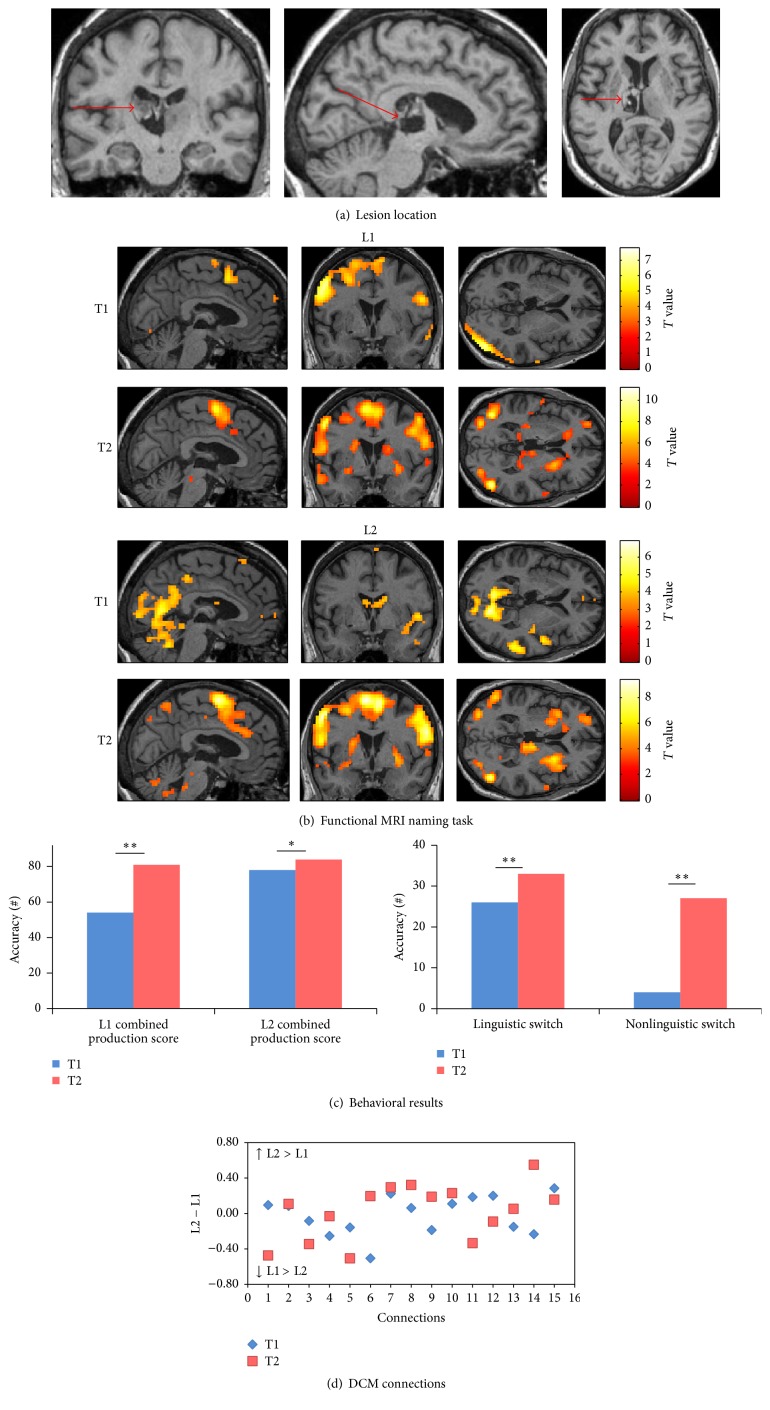
Patient 3. (a) The T1-weighted MRI image at T1 shows a hemorrhagic stroke in the left basal ganglia. (b) Pattern of brain activation in different conditions while picture naming, with an uncorrected *p* < 0.001 for the main effects. (c) Behavioral results of the combined production scores in both languages, linguistic and nonlinguistic switching scores across sessions. *∗* represents *p* value < 0.05 and *∗∗* represents *p* value < 0.001. (d) Differences between L1 strength values and L2 strength values for each single connection across sessions. (1) ACC to LIFGTri. (2) ACC to LIFGOrb. (3) ACC to LC. (4) LC to ACC. (5) LC to LIFGTri. (6) LC to LIFGOrb. (7) LIFGTri to LC. (8) LIFGTri to ACC. (9) LIFGOrb to LC. (10) LIFGOrb to ACC. (11) LIFGTri to LIFGOrb. (12) LIFGOrb to LIFGTri. (13) BTLA to LIFGTri. (14) BTLA to LIFGOrb. (15) BTLA to LC.

**Figure 6 fig6:**
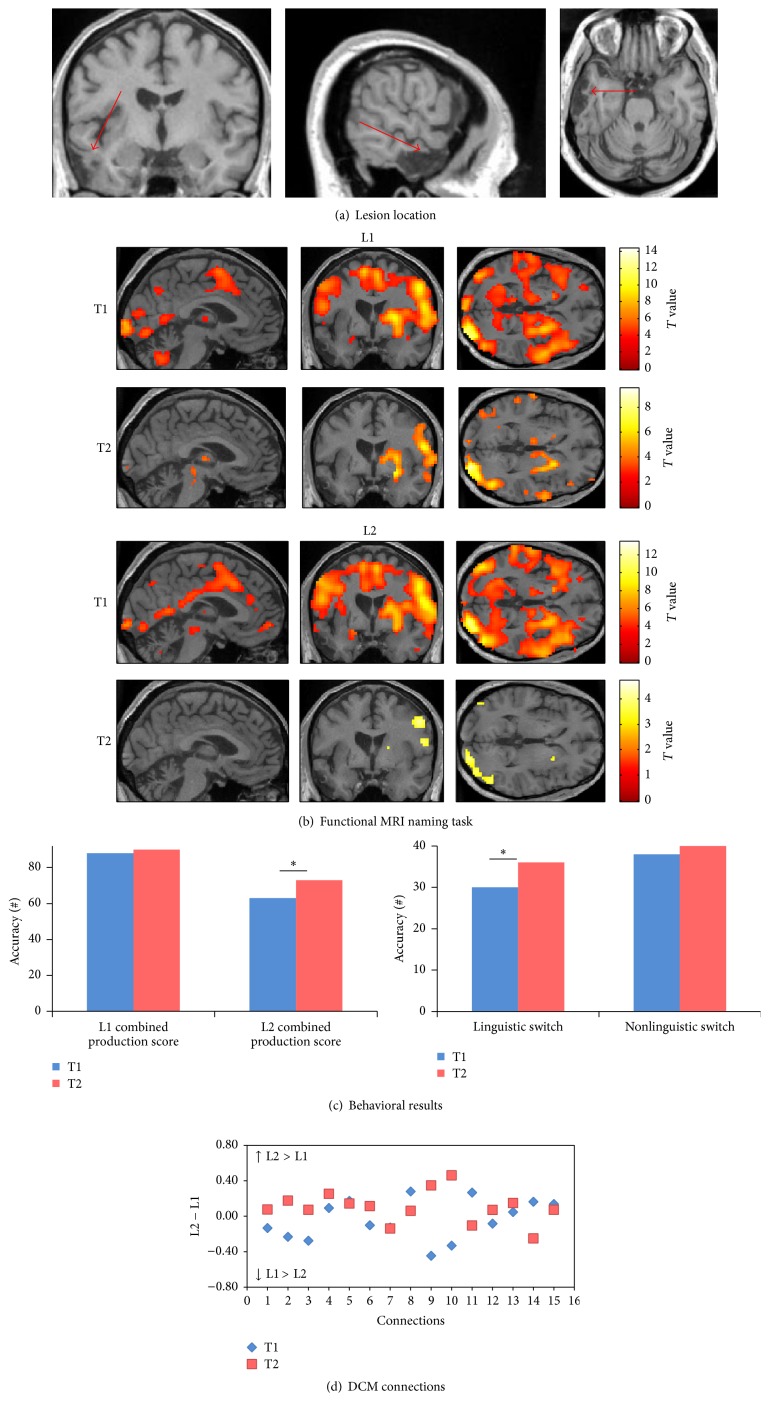
Patient 4. (a) The T1-weighted MRI image at T1 shows a left sylvian ischemic stroke. (b) Pattern of brain activation in different conditions while picture naming, with an uncorrected *p* < 0.001 for the main effects. (c) Behavioral results of the combined production scores in both languages, linguistic and nonlinguistic switching scores across sessions. *∗* represents *p* value < 0.05. (d) Differences between L1 strength values and L2 strength values for each single connection across sessions. (1) ACC to LIFGTri. (2) ACC to LIFGOrb. (3) ACC to LC. (4) LC to ACC. (5) LC to LIFGTri. (6) LC to LIFGOrb. (7) LIFGTri to LC. (8) LIFGTri to ACC. (9) LIFGOrb to LC. (10) LIFGOrb to ACC. (11) LIFGTri to LIFGOrb. (12) LIFGOrb to LIFGTri. (13) BTLA to LIFGTri. (14) BTLA to LIFGOrb. (15) BTLA to LC.

**Figure 7:**
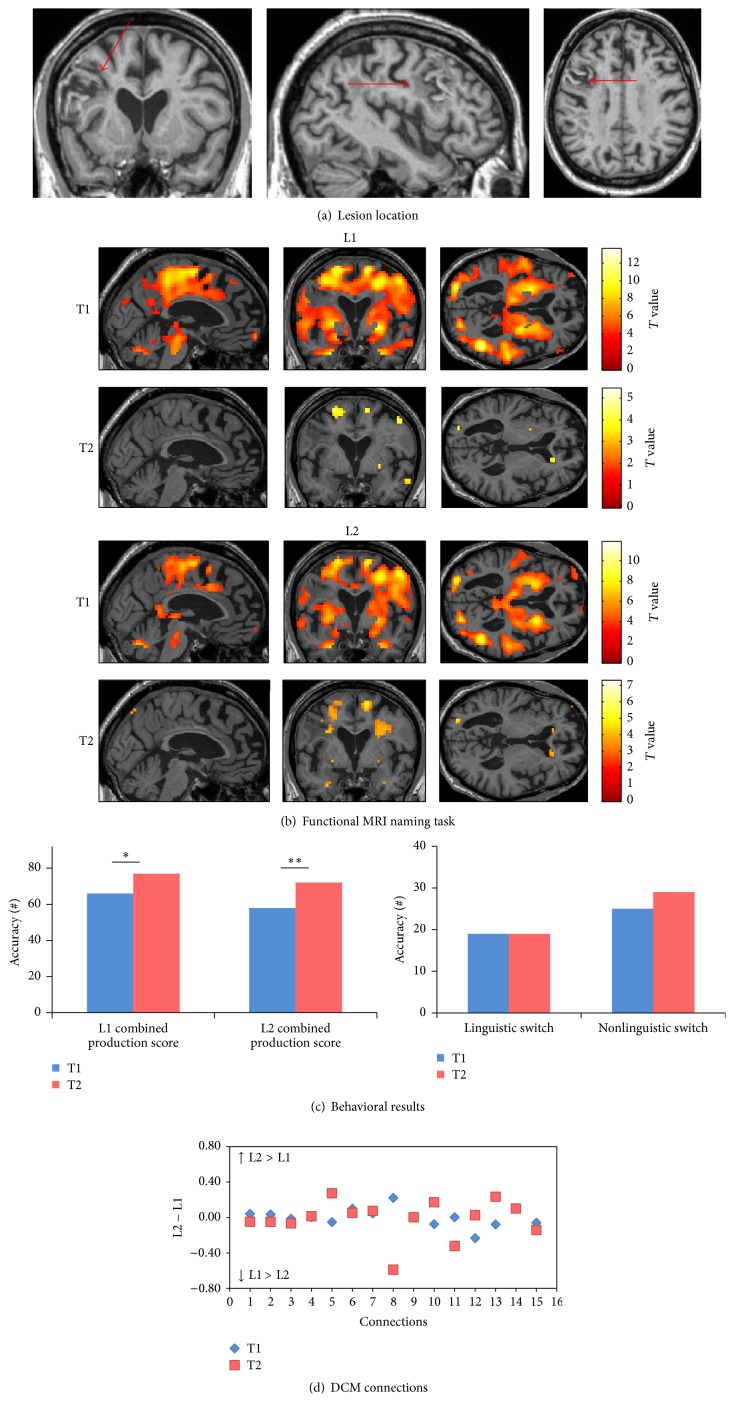
Patient 5. (a) An ischemic lesion in the left frontal, insula, and sylvian areas in the T1-weighted MRI image at T1. (b) Pattern of brain activation in different conditions while picture naming, with an uncorrected *p* < 0.001 for the main effects. (c) Behavioral results of the combined production scores in both languages, linguistic and nonlinguistic switching scores across sessions. *∗* represents *p* value < 0.05 and *∗∗* represents *p* value < 0.001. (d) Differences between L1 strength values and L2 strength values for each single connection across sessions. (1) ACC to LIFGTri. (2) ACC to LIFGOrb. (3) ACC to LC. (4) LC to ACC. (5) LC to LIFGTri. (6) LC to LIFGOrb. (7) LIFGTri to LC. (8) LIFGTri to ACC. (9) LIFGOrb to LC. (10) LIFGOrb to ACC. (11) LIFGTri to LIFGOrb. (12) LIFGOrb to LIFGTri. (13) BTLA to LIFGTri. (14) BTLA to LIFGOrb. (15) BTLA to LC.

Considering the limited number of patients and the effect of the different scanners used in this study, we primarily performed the analyses at a single-case level. Patterns of brain activation in the four different conditions (picture naming in L1 and L2 at T1: subacute phase and T2: chronic phase) for each patient are shown in the figure representing the data related to the patient (Figures 3(b) [Fig fig4] [Fig fig5] [Fig fig6]–7(b)).

Regarding the DCM analyses, in order to investigate how connection strength changes over time for single intrinsic connections within the network, the differences in the strength of connection between L1 and L2 (L2 − L1) at each session are presented in a graph for each patient. These graphs represent the pattern of difference in connection strength in language-control network while performing picture naming in L1 and L2 across time (these graphs are shown in Figures 3(d) [Fig fig4] [Fig fig5] [Fig fig6]–7(d)).

At the group level, correlation analyses were performed with aphasic patients to investigate possible correlations between the changes in connection strength (especially for the connections between control and language areas) and the changes in combined production scores for each language separately.

## 3. Results

### 3.1. General Approach

(A) We first conducted McNemar Chi-squared tests comparing language performance (GAE and picture naming scores) in L1 and L2 and control function (linguistic and nonlinguistic switch task scores) across time; (B) using DCM on fMRI, we compared the strength of connectivity within the language-control network between L1 and L2 across time at single subject level; (C) at the group level, we then performed a correlation analysis between the recovery of language production scores and the changes in the strength of connection between language and control areas. A description of the main results of the analyses is provided here, and a complete reporting of the scores and results is provided in Table 3 and Supplementary Data 1.

### 3.2. Single-Case Analyses

#### 3.2.1. Patient 1

*(A) Behavioral Scores.* At T2 (chronic phase), the combined production score showed improvement in L1 (*χ* ^2^: 12.7, *p*: 0.005) but no changes in L2. In addition, no improvement was found in linguistic and nonlinguistic switching tasks accuracy (Figure 3(c)).

*(B) Changes in Connectivity in the Language-Control Network.* For each single intrinsic connection within the network, the differences in connection strengths between L1 and L2 (L2 − L1) at each session are shown in Figure 3(d). At T1 (subacute phase), seven connection strengths were greater for L1 and eight connections had greater coupling values for L2. At T2 (chronic phase), however, the majority of connections (10 out of 15) had stronger coupling values for L1 compared to L2 (i.e., the following five connections had higher strength values in L2 at T2 (chronic phase): connections from LC to ACC, LIFGTri, and LIFGOrb, from LIFGTri to LC, and from BTLA to LIFGTri). The rest of the 15 connections had higher strength values in L1 at T2 (chronic phase). These changes in strength values indicated a globally higher connectedness inside the language-control network for L1.

#### 3.2.2. Patient 2

*(A) Behavioral Scores.* At T2 (chronic phase), the combined production score improved in both L1 (*χ* ^2^: 9.09, *p*: 0.002) and L2 (*χ* ^2^: 5.14, *p*: 0.023). However, no significant improvement was found in linguistic (*χ* ^2^: 3.2, *p*: 0.07) and nonlinguistic switching tasks (*χ* ^2^: 0.5, *p*: 0.47) (Figure 4(c)) (see Table 3 for details of the patient's performance).

*(B) Changes in Connectivity in the Language-Control Network*. Regarding the DCM analyses for each single intrinsic connection within the network, the same approach was used as for patient 1 (Figure 4(d)). Importantly, a notable change was seen in the language-control network in the pattern of differences in connection strengths between L1 and L2 from T1 (subacute phase) and T2 (chronic phase): at T1, five connections had greater strength values for L1 and 10 connections had greater strength for L2. At T2 (chronic phase), seven connections had greater strength values for L1 and eight connections had greater strength values for L2. Across time, eight connections showed different patterns of difference between L1 and L2. In particular, the connections from ACC to LIFGTri, from LIFGOrb to LIFGTri, and from ACC to LC had higher strength values for L2 compared to L1 at T2 (chronic phase), while the connections from LC to ACC, LIFGTri, and LIFGOrb, from ACC to LIFGOrb, and from LIFGTri to AB47 showed greater strength values for L1 compared to L2 at T2 (chronic phase). Although reorganization happened in the connection strengths for L1 and L2 at T2 (chronic phase), there was a similar degree of connectedness within the language-control network for L1 and L2.

#### 3.2.3. Patient 3

*(A) Behavioral Scores. *At T2 (chronic phase), the combined production score showed improvement in both L1 (*χ* ^2^: 25.07, *p* < 0.0001) and L2 (*χ* ^2^: 4.16, *p*: 0.041) at T2 (chronic phase). The patient also showed a significant improvement in both linguistic and nonlinguistic switching tasks across time (*χ* ^2^: 17.05, *p* < 0.0001 and *χ* ^2^: 21.04, *p* < 0.0001, resp.) (Figure 5(c)) (see Table 3 and Supplementary Data 1 for details of the patient's performance).

*(B) Changes in Connectivity in the Language-Control Network.* The differences between L1 and L2 (L2 − L1) in the strength of single intrinsic connections within the network are shown in Figure 5(d). The raw differences in the strength of connections within the language-control network in this patient also indicated differing patterns in the connection strengths between L1 and L2 from T1 (subacute phase) and T2 (chronic phase) in half of the connections; notably, the connections from ACC to LIFGTri and forward and backward connections between LIFGTri and LIFGOrb showed greater connection strengths for L1 compared to L2 at T2 (chronic phase). However, forward and backward connections between LC and LIFGOrb and the connection from BTLA to LIFGTri and LIFGOrb had higher connection strength values for L2 compared to L1 at T2 (chronic phase). Overall, at T1 (subacute phase), seven connections had higher strength values in L1 while at T2 (chronic phase), nine connections had higher strength values in L1. Altogether, there was a similar degree of connectedness within the language-control network for L1 and L2 at T2 (chronic phase).

#### 3.2.4. Patient 4

*(A) Behavioral Scores. *At T2 (chronic phase), the combined production score improved in L2 (*χ* ^2^: 8.16, *p*: 0.004) and no improvement was seen in L1 (already spared at T1 (subacute phase)). His performance in the linguistic switching task improved significantly (*χ* ^2^: 4.16, *p*: 0.041) and his nonlinguistic switching performance was spared at T1 (subacute phase) (Figure 6(c)).

*(B) Changes in Connectivity in the Language-Control Network.* At the single intrinsic connection level, the differences between L1 and L2 (L2 − L1) in strength of single intrinsic connections within the network for each session are shown in Figure 6(d). At T1 (subacute phase), around half of connections had higher strength values for L1 (eight out of 15), while at T2 (chronic phase), only three connections had greater values for L1 (i.e., connection from LIFGTri to LC, LIFGTri to LIFGOrb, and BTLA to LIFGOrb) and the rest of the connections showed higher coupling values for L2. These changes indicated a globally higher connectedness inside the language-control network for L2 at T2 (chronic phase).

#### 3.2.5. Patient 5

*(A) Behavioral Scores.* The combined production score improved in both L1 (*χ* ^2^: 9.09, *p*: 0.002) and L2 (*χ* ^2^: 12.07, *p*: 0.0005) at T2 (chronic phase), although the patient still made several language switching errors. However, no improvement was seen in the linguistic and nonlinguistic switching task performances (Figure 7(c); more details can be found in Table 3 and Supplementary Data 1).

*(B) Changes in Connectivity in the Language-Control Network.* At the single intrinsic connection level, the differences between L1 and L2 (L2 − L1) in the strength of single intrinsic connections within the network for each session are shown in Figure 7(d). Importantly, several connections showed inverse patterns between T1 (subacute phase) and T2 (chronic phase); that is, four connections (from ACC to LIFGTri, ACC to LIFGOrb, LIFGTri to ACC, and LIFGTri to LIFGOrb) had higher strength values for L1 at T2 (chronic phase), and four connections (from LC to LIFGTri, LIFGOrb to ACC, LIFGOrb to LIFGTri, and BTLA to LIFGTri) had greater strength values for L2 at T2 (chronic phase). As with the changes seen in patients 2 and 3, there was a similar connectedness within the language-control network in L1 and L2 at T2 (chronic phase).

### 3.3. Group Analyses of fMRI and DCM Analyses

#### 3.3.1. FMRI Results

For the aphasic patients, the patterns of activation at each session of picture naming in L1 and L2 were presented for each patient separately; a threshold of uncorrected *p* < 0.001 was selected to visualize the main effects (Figures 3(b) [Fig fig4] [Fig fig5] [Fig fig6]–7(b)). As our main aim of the fMRI study was to perform DCM analysis based on a previously published model, we did not statistically compare the activations in the different conditions.

#### 3.3.2. DCM Results

*(C) Correlation Analysis between Language Production Recovery and Changes in the Strength of Connection. *At the group level, in the aphasic patients, the changes in the strength of intrinsic connections between language and control areas (specifically between ACC, LC, and LIFGOrb from control subnetwork to LIFGTri in language subnetwork) were implemented to correlate with the changes in combined production scores.

In the aphasic patients, we found a significant correlation between changes in the combined production scores in L1 and changes in the strength of connection from ACC to LIFGTri (while performing picture naming in L1) (Spearman's rho: 0.921, *p*: 0.026). Moreover, changes in the combined productions score in L2 were negatively correlated to the changes in the strength of connections from LIFGTri to LIFGOrb (Spearman's rho: −0.900, *p*: 0.037).

### 3.4. Supplementary Analyses of DCM

To better compare the changes in the number of connections with higher strength values between the improved versus unimproved language across time, we concatenated the data of patients 1 and 4 (who improved language production in only one language). This combined analysis showed a higher number of connections in the improved language at T2 (chronic phase) (*χ* ^2^: 4.44, *p*  0.035).

## 4. Discussion

Using a longitudinal design, we examined language production recovery in five late bilingual patients suffering from poststroke aphasia at subacute and chronic phases following a stroke. From three weeks to four months following a stroke, (A) we monitored modifications in language and control performance to identify whether language recovery was linked with the recovery of control functions. Moreover, (B) using a DCM approach, we examined how the interconnections between language and control areas changed with the recovery of language production, and (C) we then investigated the possible correlation between changes in language production performances and changes in the strength of each single connection within language-control network across time.

Considering the changes in the combined production scores, three of our five patients recovered in both L1 and L2 (patients 2, 3, and 5), one patient recovered in L1 (patient 1), and one (patient 4) in L2 only (the latter patient already had a high accuracy score in L1 at the subacute phase). Two patients (patient 3 with recovery in both languages and patient 4 with recovery in L2) showed improvement in language-control functions (Table 4). No decrease in control functions was observed among the patients.

Descriptive analyses of the DCM suggested a relationship between the pattern of recovery of language production and changes in the strength of connections across time. In patient 1, who recovered only L1 production score across time, the majority of connections within language-control network (10 out of 15 connections) had higher connection strength values at the chronic phase, indicating a higher connectedness within the language-control network while picture naming in L1. The similar pattern of changes in the connectedness within language-control network took place in patient 4 who recovered only L2 (i.e., at the chronic phase he showed higher connectedness within language-control network while picture naming in L2). In these two patients with recovery of only one language, combined analyses revealed that improvement in production score in one language was associated with an increase in the number of connections with higher strength values at T2 (chronic phase) while performing the task in that specific language. In addition, showing a similar pattern of changes in the language-control network connectedness, patients 2 and 5 at T1 (subacute phase) in the majority of connections had higher coupling values for picture naming in L2, while at T2, the coupling values of 7 connections were higher in L1 and 8 connections had higher coupling values in L2 task. Also, patient 3 showed higher coupling values for the majority of connections for L1 picture naming at T1 (subacute phase), while at T2, the coupling values of 7 connections were higher in L1 and 8 connections had higher coupling values in L2 task. Taken together, in patients 2, 3, and 5 who recovered both L1 and L2, a redistribution of the connection strength occurred across time; the strength of the connections between language and control areas was similarly distributed at T2 (chronic phase) over the network during picture naming in L1 and L2. In the control group with main L2 exposure and usage in daily life, a higher connectedness was seen within the network for L1 compared to L2 (see Supplementary Data 2 and 3). We will discuss each of these results in turn.

Although the role of control functions in the recovery of bilingual aphasia has been suggested in several studies [8, 9], in our patients, at the behavioral level, the improvement in language-control functions alone could not explain their patterns of language recovery. The observed pattern of language and control recovery does not directly support Paradis' statement that when language-control function is intact, one can expect a parallel recovery of languages, and in the presence of language-control problems one may expect the weaker language in the premorbid stage to be impaired [34]. However, Green and Abutalebi [35] suggest that, along with premorbid proficiency, languages that were mostly used following stroke may become more proficient and easily manageable, especially in the case of reduced resources for controlling the use of two languages.

Our findings on the changes in the differences in connectedness between L1 and L2 within the language-control network are in line with the results of Abutalebi et al. [9], in the case of a bilingual aphasic patient for whom the language which recovered better showed increased connections between language and control networks. Our DCM results support a role for language-control interconnections in language recovery in bilingual aphasic patients [9, 10, 35] and are in line with the “dynamic view” of language production, which posits that patterns of language recovery are related to alterations in language control. Interestingly, for patients in whom both languages recovered (patients 2, 3, and 5), the two languages were connected to the control system to the same extent. Additionally, when one language recovered better, there was a greater engagement of language-control interconnections in this language. Previous studies of the association between global patterns of brain connectivity and the recovery of language functions have suggested that decreased functional connectivity between anterior and posterior areas of the default mode network (DMN) is associated with cognitive impairment. Accordingly, therapy-induced increases in functional connectivity between anterior and posterior areas of the DMN have been reported in a group of chronic monolingual aphasic patients [36]. In a further study, Sebastian and colleagues [37] evaluated the recovery of naming functions from acute to chronic phase and showed that the degree of functional connectivity between language-specific areas in both hemispheres was important for optimal recovery of naming functions.

Furthermore, our results suggest that a change in connection strength from ACC to LIFGTri during picture naming in L1 was associated with L1 recovery; the coupling between these two areas became stronger when L1 recovered. LIFGTri, along with the LIFG pars opercularis (BA44), is known to be involved in different steps of language production [38], namely, in syntactic encoding [39], speech praxis [40], and verb retrieval [41]. ACC is known to be involved in conflict and error monitoring, including domain-general control functions in healthy populations [42]. In the normally functioning bilingual brain, ACC, in connection with the prefrontal cortex, is a component of the circuit involved in inhibiting interference from the nontarget language [1, 18, 43] while in a healthy brain, this interference is caused mainly by the more proficient language (usually L1); our results could be explained by the fact that in the presence of language and control dysfunction (e.g., following a stroke), conflicts may arise between L1 and L2 even in the case of different proficiencies. Therefore, a higher engagement of the circuit between ACC and LIFGTri could possibly facilitate performance in the recovery of L1 by blocking the interference of information from L2.

Analyses of the changes in connectivity strengths across time suggest that, in patients with L2 recovery (four out of five patients), the connection from LIFGTri to LIFGOrb becomes weaker for L2 compared to L1. This finding is also supported by the result of a correlation analysis showing that the recovery of combined production scores in L2 negatively correlates with changes in the strength of connection from LIFGTri to LIFGOrb. In other words, when L2 recovers, the coupling from LIFGTri to LIFGOrb decreases. These two regions are strongly anatomofunctionally interconnected as subregions of the inferior frontal gyrus [40]. LIFGTri is selected as the main language production area and LIFGOrb is a part of language-control network, which is involved in both language production and language-control processes (lexical semantic processes along with LIFGTri and selecting among lexical competitors) [44, 45]. The reason for the decrease of coupling from LIFGTri to LIFGOrb in L2 production could be explained by the Revised Hierarchical Model of lexical and conceptual representation in the bilingual brain [46, 47]. In this psycholinguistic model the conceptual system is common across languages, even in the less proficient L2. However, L1 is hypothesized to have privileged access to the conceptual system, favored by a strong connection between the areas involved in lexical and semantic processing (resp., LIFGTri and LIFGOrb in our study). Hence, a weaker connectivity between these two areas may help in the process of L2 recovery.

The present study suffers from several limitations. First, we could not implement certain language and control tasks since they were too demanding for aphasic patients, especially in the acute and subacute phases. A comprehensive evaluation of language and control would have helped better understand the possible correlation between the recovery of control functions and the recovery of language performance following a stroke. Even though we narrowed our selection of tasks to a limited series of evaluation materials, half of the initially recruited patients dropped out of the second session of the study. Another limitation of this study was the small number of included patients; their results may not be easily applicable to all bilingual aphasic patients. In addition, we could not control the age of the patients in the study (the age of patients ranged from 49 to 79 years old). It is known that the age factor can affect behavioral performance, functional brain activity, and connectivity within brain areas as a result of alteration in neuronal activity and connectivity in aging brain [48, 49]. However, as the design is mainly within-subject the age does not seem to affect importantly the results. In addition, regressing out the effect of age from the analyses would not let any significant results due to the small sample size. Moreover, the use of different MRI scanners in this study restricted us in carrying out a direct group comparison of brain activation in different conditions. Finally, as obtaining an accurate measure of premorbid proficiency following their stroke was impossible, the evaluation of premorbid second language proficiency was restricted to a detailed questionnaire filled in by a family member or the patient himself.

It worth noting that, in the present study, only three patients followed language therapy sessions and the therapy was computer assisted to improve lexical access and in turn improve naming performances. One patient (patient 1) received therapy in his L1 (French) and he then improved in L1 production. This lack of improvement in L2 could be explained by the very low L2 usage and immersion by this subject. It has been previously suggested by Edmonds and Kiran [50] that the effect of therapy in less mastered language is more likely to transfer to the untreated language as the subject is more relied on borrowing word from the more proficient language. Another explanation of the absence of transfer of the effect of language therapy in L1 to the untreated L2 is the fact that he did not show improvement in control functions across time [8]. Patient 3 received therapy in her L2 (French), which she has been used and was immersed in an equal level as her L1 since 53 years ago. She improved in both L1 (Italian) and L2 across time. Her improvement in both languages can be explained by high immersion in both languages as well as improvement in the cognitive control functions. Patient 5 attended to a limited number of therapy sessions (10 sessions) in his L1 (French) and he improved in both L1 and L2 (English). In this patient, the recovery of both languages cannot be explained by the choice of therapy or the pattern of changes in cognitive control functions. Therefore, no consistent pattern of the effect of therapy and possible cross language transfer of the effect of therapy was found. Therefore, our interpretation of these results is not based on the language therapy. Moreover, because of the timing of the study sessions (at three weeks and around four months following the stroke), the process of spontaneous recovery should be still ongoing [6, 23]. Accordingly, this recovery takes place as the result of a combination of spontaneous recovery and language therapy.

## 5. Conclusion

Taken together, our findings supply additional evidence that the engagement of the interconnected language-control network is crucial for the recovery of languages. Furthermore, we suggest that L1 recovery is improved by increased connectivity between ACC and LIFGTri, which prevents conflicts from the second language. However, L2 recovery requires a decrease in connectivity from LIFGTri to LIFGOrb in order to decrease the automatic activation of the L1 lexical system, which, according to the Revised Hierarchical Model, has stronger links with the conceptual system.

## Supplementary Material

In order to support data included in the main text, additional data is provided in Supplementary Material in three parts. Supplementary data 1 includes detailed information on patients' scores in General Aphasia Evaluation and Picture naming task. Supplementary data 2 includes information on control subjects as well as the results of DCM analysis on this subjects. Supplementary data 3 shows the pattern of brain activation whilst picture naming in L1 and L2 in control subjects.

## Figures and Tables

**Table 1:** Assessment of premorbid L2 proficiency.

	Subject 1	Subject 2	Subject 3	Subject 4	Subject 5
First language (L1)	French	Italian	Italian	French	French
Second language (L2)	English	French	French	English	English
AoA (y/o)	10	24	10	14	18
Lived in region speaking L2	0	41	53	>1	>1
*Family*					
First language of mother	French	Italian	Italian	French	Swiss German
Language spoken with mother	French	Italian	Italian	French	French
First language of father	French	Italian	Italian	French	French
Language spoken with father	French	Italian	Italian	French	French
First language of partner	French	French/Italian	Italian	French	—
Language spoken with partner	French	Italian	French	French	—
*Childhood (<7 y/o)*					
Language taught in school	French	Italian	No info.	French	French
Language spoken with peers at school	French	Italian	No info.	French	French
Language spoken with family	French	Italian	Italian	French	French
*Present*					
Spoken at work	15% L1, 85% L2	100% L2	75% L2, 25% L1	50% L1, 50% L2	50% L1, 50% L2
Watching TV/listening to radio	100% L1	100% L1	50% L1, 50% L2	50% L1, 50% L2	75% L1, 25% L2
Speaking with friends	90% L1, 10% L2	50% L1, 50% L2	50% L1, 50% L2	L1	50% L1, 50% L2
Reading books	50% L1, 50% L2	50% L1, 50% L2	50% L1, 50% L2	50% L1, 50% L2	100% L1
Mental arithmetic	100% L1	75% L1, 25% L2	50% L1, 50% L2	L1	100% L1
*Self-evaluation of L2 (0–100)*					
Speaking	100	95	100	55	80
Writing	98	15	100	75	50
Comprehension	100	95	100	80	89
Reading	100	75	100	35	70

**Table 2:** Demographic data.

	Subject 1	Subject 2	Subject 3	Subject 4	Subject 5
Age	61	65	63	49	79
Gender	M	F	F	M	M
Scholarity (years)	16	12	12	18	16
Lesion site	Left frontotemporal	Left frontobasal area	Left basal ganglia	Left sylvian	Left frontal-insula-sylvian
Lesion etiology	Ischemic + hemorrhagic stroke	Epidural hematoma	Hemorrhagic stroke	Ischemic stroke	Ischemic stroke
Time after stroke at T1 (days)	34	21	64	7	21
Time after stroke at T2 (days)	120	118	166	110	135
Language therapy between T1 and T2					
Language of therapy	L1	L2	L2	—	L1
Number & duration of therapy sessions	25 sessions (45 min./session)	1 session (30 min.)	41 sessions (45 min./session)	—	10 sessions (45 min./session)
Type of therapy	CAT CIAT	CAT	CAT	—	CAT

CAT: computer assisted therapy for anomia to improve lexical access.

CIAT: constraint induced aphasia therapy.

**Table 3:** General aphasia evaluation, picture naming, and control functions scores at T1 and T2.

Task	Session	General aphasia evaluation (GAE)											Picture naming (/40)	Combined production score (/92)	Language control	
		Object naming (/20)	Series (/6)	Verbal fluency (/3)	Repetition (/13)	Yes/no questions (/20)	Pointing (/5)	Following commands (/8)	Reading commands (/11)	Description (/10)	Production score (GAE) (/52)	Total (/96)			Linguistic switch (/40) (number of switching errors)	Nonlinguistic switch (/40) (number of switching errors)
Subject 1	T1 L1	10	6	0	13	13	4	4	2	10	39	62	20	59	9 (7)	15 (13)
	T1 L2	4	4	0	13	16	4	1	3	10	31	55	1	32		
	T2 L1	13	6	0	13	17	5	6	8	8	40	76	28	68	11 (5)	16 (24)
	T2 L2	4	4	0	13	16	4	1	3	10	31	55	2	33		

Subject 2	T1 L1	16	6	0	13	16	5	4	9	10	45	79	26	71	26 (5)	38 (1)
	T1 L2	19	6	1	9	20	5	5	7	10	45	82	28	73		
	T2 L1	19	6	1	13	18	5	5	9	10	49	86	33	82	31 (4)	40
	T2 L2	20	6	0	13	16	5	5	9	10	49	84	31	80		

Subject 3	T1 L1	18	6	0	13	18	5	8	7	10	47	85	7/14	54/66	14 (11)	4 (15)
	T1 L2	18	6	0	13	20	5	7	4	10	47	83	31	78		
	T2 L1	20	6	1	13	20	5	8	11	10	50	94	31	81	33 (2)	27 (8)
	T2 L2	20	6	0	13	20	5	8	11	10	49	93	35	84		

Subject 4	T1 L1	18	6	2	13	16	5	8	11	10	49	89	39	88	30 (0)	38 (2)
	T1 L2	11	6	0	13	20	4	8	10	6	36	78	27	63		
	T2 L1	20	6	3	13	18	5	8	11	10	59	94	38	90	36 (1)	40 (0)
	T2 L2	12	6	3	13	18	4	8	11	8	42	83	31	73		

Subject 5	T1 L1	18	6	0	13	18	5	2	9	10	47	81	19	66	19 (17)	25 (3)
	T1 L2	12	4	0	13	20	5	4	8	10	39	76	19	58		
	T2 L1	18	6	0	13	18	5	4	11	10	47	85	30	77	19 (13)	29 (4)
	T2 L2	16	6	0	13	16	5	4	11	10	45	81	27	72		

**Table 4:** Summary of the recovery patterns.

Subject	L1-combined production	L2-combined production	Linguistic switch	Nonlinguistic
Patient 1	↑	→	→	→
Patient 2	↑	↑	→	→
Patient 3	↑	↑	↑	↑
Patient 4	→	↑	↑	→
Patient 5	↑	↑	→	→
