# Supplementary material for: The Role of the Cognitive Control System in Recovery from Bilingual Aphasia: A Multiple Single-Case fMRI Study
Source: Neural Plast. 2016 Nov 14;2016:8797086. doi: 10.1155/2016/8797086 (PMC5124691; doi:10.1155/2016/8797086)
Supplement: Supplementary file 1 — In order to support data included in the main text, additional data is provided in Supplementary Material in three parts. Supplementary data 1 includes detailed information on patients' scores in General Aphasia Evaluation and Picture naming task. Supplementary data 2 includes information on control subjects as well as the results of DCM analysis on this subjects. Supplementary data 3 shows the pattern of brain activation whilst picture naming in L1 and L2 in control subjects. [file 8797086.f1.pdf]

Supplementary Data 1:

Details on general aphasia evaluation and picture naming score:

Patient 1

General aphasia evaluation (GAE) at T1 showed impairment in object naming (several semantic paraphasias), verbal fluency, yes/no questions and following oral and written semi-complex and complex commands in both L1 and L2. At T2, the scores remained stable for L2, however improvement was seen mainly in yes/no questions and following oral and written semi-complex and complex commands. Mcnemar chi-squared test over the total GAE score showed a significant improvement in the total GAE score of L1 (X^2: 12.7, P: 0.005). In L2 no improvement was found in the total GAE score. Picture naming during fMRI acquisition showed several errors in both L1 and L2 at T1 (21 of 40 images correctly named in L1 while only one picture correctly named in L2, he switched repeatedly to L1 and produced 5 semantic errors).

At T2, there was a significant improvement in picture naming in L1 (X^2: 7.11, p: 0.007), however, no improvement was found in picture naming in L2.

Patient 2

General aphasia evaluation at T1, showed slight impairment in object naming, verbal fluency, yes/no questions and following oral semi-complex and complex commands in L1 and slight impairment in verbal fluency, word/phrase repetition and following oral and written semi-complex and complex orders L2. At T2, the scores remained stable for both languages in all subtests except for an improvement in object naming in L1 and word and phrase repetition in L2. The total GAE score showed improvement in the total GAE score of L1 (X^2: 5.14, P: 0.023). In L2 no improvement was found in the total GAE score. Picture naming during fMRI acquisition showed low accuracy score in both L1 and L2 at T1 (26 of 40 images correctly named in L1 and 28 of 40 picture correctly named in L2).

At T2, there was an improvement in picture naming in L1 (X^2: 5.14, p: 0.02), while no improvement was found in picture naming in L2.

Patient 3

General aphasia evaluation at T1, showed a generally good performance in all the subtests except a slight impairment in object naming and moderate impairment in verbal fluency and following written semi-complex and complex commands in both L1 and L2. At T2, her performance improved generally in both L1 and L2 (except for the absence of improvement in verbal fluency in L2). Mcnemar chi-squared

test over the total GAE score showed a significant improvement in the total GAE score of both L1 (X^2: 7.11, P: 0.007) and L2 (X^2: 8.1, P: 0.004).

Picture naming during fMRI acquisition showed a significant improvement at T2 in picture naming in L1 (X^2: 5.3, p: 0.02), however, no improvement was found in picture naming in L2 across time.

Patient 4

General aphasia evaluation at T1 in L2 showed impairment in object naming, verbal fluency and description, while L1 performance in all subtests were spared except for one error in object naming and two errors in yes/no questions. His performance in the total GAE score was stable in both L1 and L2 between T1 and T2; L1 (X^2: 3.2, P: 0.07) and L2 (X^2: 3.2, P: 0.07).

Picture naming during fMRI acquisition showed an impaired performance in L2 at T1 (mainly due to no responses), while picture naming in L1 was performed quite correctly. At T2, no improvement was found in picture naming in L2 (X^2: 2.25, P: 0.13).

Patient 5

General aphasia evaluation at T1, showed a slight impairment in object naming (only one language switching error), impaired verbal fluency, series and impairment in following auditory and written commands in L1. However, in L2, he showed impairment in object naming, verbal fluency and following auditory and written commands. At T2, his performance improved in written command in both L1 and L2. He also showed improvement in object naming in L2, however he performed more errors in yes/no questions in L2. Mcnemar chi-squared test over the total GAE score showed no significant improvement in the total GAE score of both L1 (X^2: 2.25, P: 0.13) and L2 (X^2: 3.2, P: 0.07).

Picture naming during fMRI acquisition showed an impaired performance in both L1 and L2 at T1 (he had several switching errors in both languages). At T2, picture naming improved in both L1 and L2 (X^2: 17.05, P<0.0001 and X^2: 6.12, P: 0.013, respectively).

Supplementary Data 2:

Control subjects:

Control subjects recruitment:

We recruited six healthy subjects matched by age (65.6±8.5), education and AoA (18.6±4.3) however one subject was removed from the analyses as he did not meet our criteria for DCM analyses (i.e. lack of activation in one of the ROIs). The control subjects all speak French as their second language; two subjects speak Italian as L1, two speak German and one speaks Spanish as L1. In all control subjects, their main language usage and exposure at work and with family and friends is L2.

Control subjects were evaluated in a single session using behavioral language control tasks (linguistic and non-linguistic switching) and fMRI picture naming tasks. The MRI recording was performed in the scanner 2 for all the control subjects.

FMRI data analyses:

For the control group, brain activation during picture naming in L1 and L2 was shown using Fixed Effect Analyses (FFX) of the activation with a threshold of $p<0.05$ FWE corrected (Supplementary figure 1 a). The coordinates of the activated areas are summarized in supplementary Data 3.

DCM results:

In the control subjects, the differences in the average connection strength for each connection across subjects are shown in Supplementary figure 1 b. In the control group, for each single connection in the network, the difference in the average connection strength across subjects is shown in Supplementary figure 1 b. In this group average, most of the connections (9 of 15) have greater strength values for L1 compared to L2; i.e., higher connectedness within the language-control network for L1 compared to L2.

Supplementary Data 3:

Pattern of activation in control subjects: Fixed Effect analyses of fMRI data shows the pattern of brain activation in our control group while picture naming in L1 and L2. A significance threshold of p<0.05 (FWE corrected) was applied.

	Coordinates (x,y,z)			T	p (FWE)	Brodman area	MNI-aal	Neuromorphometric	Side
Picture naming L1	-33	-85	-8	21.34	0.000	18, 19	Inf. Occipital	Inf. Occipital gyrus	Left
	-51	-10	34	19.41	0.000	4, 6	Post central	Postcentarl gyrus	Left
	33	-85	-5	19.05	0.000	18, 19	Inf. Occipital	Inf. Occipital gyrus	Left
	-27	-5	-4	13.09	0.000	Putamen	Putamen	Putamen	Left
	-30	26	4	7.86	0.000	13	Insula	Ant. Insula	Left
	-9	-16	4	8.53	0.000	Medial dorsal nucleus	Thalamus	Thalamus	Left
	51	-4	31	19.07	0.000	6	Post central	Precentral gyrus	Right
	60	-1	22	18.41	0.000	6, 4	Post central	Precentral gyrus	Right
	21	11	1	9.65	0.000	Putamen	Putamen	Putamen	Right
	12	-16	4	7.74	0.000	Medial dorsal nucleus	Thalamus	Thalamus	Right
	45	20	22	6.44	0.000	9	Inf. Frontal Tri	Inf. Frontal Op	Right
	48	-31	49	5.75	0.000	40	Post central	Supramarginal gyrus	Right
	21	-58	7	4.92	0.010	30	Calcarine	Precuneus	Right
	51	14	-11	4.95	0.041	13	Temporal pole sup.	Temporal pole	Right
	36	29	1	4.57	0.046	13	Inf. Frontal Tri	Inf. Frontal Orb	Right
	0	-22	-17	6.43	0.000	Red Nucleus	Unknown	Left ventral	
Picture naming L2	-33	-85	-8	20.38	0.000	19	Inf. Occipital gyrus	Inf. Occipital gyrus	Left
	-36	26	1	7.31	0.000	13	Inf. Frontal Tri	Frontal operculum	Left
	-27	-4	-5	7.14	0.000	Putamen	Putamen	Putamen	Left
	-21	14	-2	6.65	0.000	Putamen	Putamen	Putamen	Left
	-9	-16	4	5.9	0.000	Medial dorsal nucleus	Thalamus	Thalamus	Left
	-6	-	-	4.95	0.009	Red nucleus	Para	ventral DC	Left

		25	14				hippocampus		
	-6	-73	13	4.63	0.032	18, 30	Calcarine	Calcarine	Left
	36	-82	-5	20.34	0.000	18, 19	Inf. Occipital gyrus	Inf. Occipital gyrus	Right
	27	2	-5	18.75	0.000	Putamen	Putamen	Putamen	Right
	6	-22	-17	5.74	0.000	Red nucleus	Unknown	ventral DC	Right
	39	20	25	5.44	0.001	9	Inf. Frontal Tri	Middle frontal gyrus	Right
	9	-16	4	5.1	0.004	Medial dorsal nucleus	Thalamus	Thalamus	Right
	12	-82	40	4.67	0.027	19, 18	Cuneus	Cuneus	Right

Supplementary figure 1. Control group

a. Functional MRI Naming task

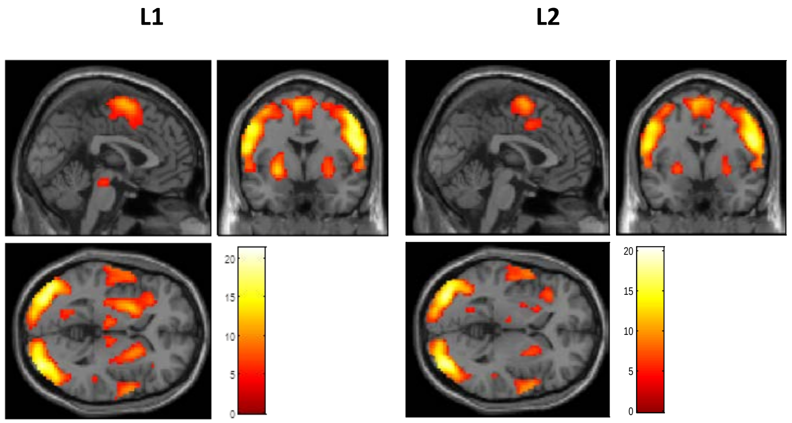

b. DCM connections

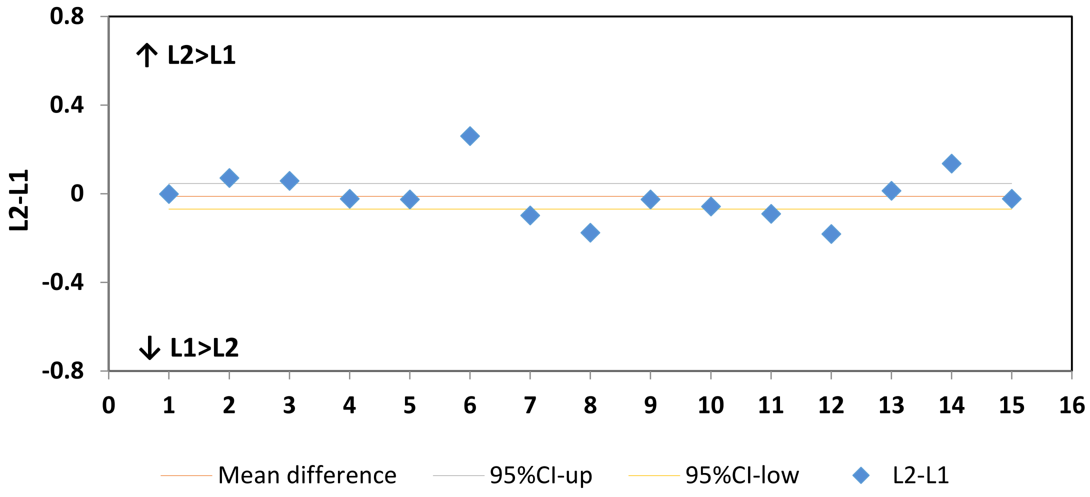

1. ACC to LIFGTri
2. ACC to LIFGOrb
3. ACC to LC
4. LC to ACC
5. LC to LIFGTri
6. LC to LIFGOrb
7. LIFGTri to LC
8. LIFGTri to ACC
9. LIFGOrb to LC
10. LIFGOrb to ACC
11. LIFGTri to LIFGOrb
12. LIFGOrb to LIFGTri
13. BTLA to LIFGTri
14. BTLA to LIFGOrb
15. BLTA to LC
